# Photobleaching Kinetics and Time-Integrated Emission of Fluorescent Probes in Cellular Membranes

**DOI:** 10.3390/molecules190811096

**Published:** 2014-07-29

**Authors:** Daniel Wüstner, Tanja Christensen, Lukasz M. Solanko, Daniel Sage

**Affiliations:** 1 Department of Biochemistry and Molecular Biology, University of Southern Denmark, DK-5230 Odense M, Denmark; E-Mails: tanjac@bmb.sdu.dk (T.C.); lsolanko@gmail.com (L.M.S.); 2 Biomedical Imaging Group, Ecole Polytechnique Fédérale de Lausanne (EPFL), CH-1015 Lausanne, Switzerland; E-Mail: daniel.sage@epfl.ch

**Keywords:** photophysics, kinetics, autofluorescence correction, cholesterol, BODIPY, transport

## Abstract

Since the pioneering work of Hirschfeld, it is known that time-integrated emission (TiEm) of a fluorophore is independent of fluorescence quantum yield and illumination intensity. Practical implementation of this important result for determining exact probe distribution in living cells is often hampered by the presence of autofluorescence. Using kinetic modelling of photobleaching combined with pixel-wise bleach rate fitting of decay models with an updated plugin to the ImageJ program, it is shown that the TiEm of a fluorophore in living cells can be determined exactly from the product of bleaching amplitude and time constant. This applies to mono-exponential bleaching from the first excited singlet and/or triplet state and to multi-exponential combinations of such processes. The TiEm can be used to correct for illumination shading and background autofluorescence without the need for fluorescent test layers or separate imaging of non-stained cells. We apply the method to simulated images and to images of cells, whose membranes were labelled with fluorescent sterols and sphingolipids. Our bleaching model can be extended to include a probability density function (PDF) of intrinsic bleach rate constants with a memory kernel. This approach results in a time-dependent bleach rate coefficient and is exemplified for fluorescent sterols in restricted intracellular environments, like lipid droplets. We show that for small deviations from the classical exponential bleaching, the TiEm of decay functions with rate coefficients remains largely independent of fluorescence lifetime and illumination, and thereby represents a faithful measure of probe distribution.

## 1. Introduction 

Live-cell fluorescence microscopy has become a quantitative branch of life sciences, often complementing ‘omics’ approaches such as proteomics and lipidomics by providing direct access to spatial information about biomolecules in living cells. This is a consequence of highly specific labelling techniques, improved microscope instrumentation and advanced digital imaging devices developed over the last two decades. Quantification of organelle-associated fluorescence of a given probe by image analysis of acquired fluorescence recordings has become a standard method in many laboratories. In particular quantitative co-localization analysis is one of the most important tools for studying membrane traffic in living cells. To determine intracellular protein destinations, a protein of interest, like a certain membrane receptor, can be expressed as a green fluorescent protein (GFP)-construct in a mammalian cell line or in model organisms, like *Caenorhabditis elegans*, and its distribution is compared to that of characteristic organelle markers. These markers are typically proteins residing in the investigated organelle and are tagged with another GFP-color variant. For example, different rab-GTPases expressed as fluorescent protein constructs have been used to distinguish various endosome populations in living cells and in nematodes [1,2]. Protein trafficking can be also studied in by pulse-chase experiments of antibody-tagged protein variants, in which a particular epitope is genetically introduced into the protein of interest [3,4] or, in case of studying endocytosis, by using fluorescent ligands of endocytic receptors [5]. As alternative organelle markers, lipid analogs have been introduced. For example, the fluorescent ceramide N-(7-nitrobenz-2-oxa-1,3-diazol-4-yl-aminocaproyl)-D-*erythro*-sphingosine (C6-NBD-Cer) is used as vital stain of the Golgi apparatus, and more specifically, the trans-Golgi-network (TGN) in living and fixed cells, though it labels to some extent also the nuclear envelope and the endoplasmic reticulum (ER) [6,7]. The advantage of fluorescent lipid markers is the fact, that one can label any cell easily without the need for transfection with an organelle-resident intrinsically fluorescent protein. For example, retrograde trafficking kinetics of TGN38 to the TGN stained with C6-NBD-Cer have been studied by quantitative immunofluorescence microscopy [3]. Moreover, para-Golgi localization of the endocytic recycling compartment (ERC) containing fluorescent transferrin and sterols could be documented by two-color laser scanning or wide field microscopy with C6-NBD-Cer as marker for the proximate TGN in various cell types [3,8,9,10]. Fluorescent lipid probes, like C6-NBD-tagged sphingomyelin (C6-NBD-SM) have also been used as non-specific membrane marker, reporting about overall membrane flow and recycling [11,12,13]. Similarly, the hydrophobic membrane probe trimethylammonium diphenylhexatriene (TMA-DPH) is not only used as membrane viscosity probe with preferred orientation towards the bilayer-water interface [14], but also for studying intracellular membrane flow as well as lipid order in model and cell membranes by fluorescence polarization spectroscopy and microscopy [15,16,17,18]. In addition, fluorescent lipid analogs mimicking certain phospho- and sphingolipids have been used to study endocytic lipid sorting and to determine lipid trafficking routes in polarized epithelial cells [19,20,21,22]. Finally, a great deal of effort has been spent to determine intracellular trafficking routes of cholesterol using fluorescent cholesterol analogs [23,24]. Here, either cholesterol with a minimally perturbing reporter moiety, such as BODIPY-tagged cholesterol (BChol) has been applied [25,26], or intrinsically fluorescent sterols, like the yeast sterol dehydroergosterol (DHE) or the related cholestatrienol are often used [27]. While the first gives much better fluorescence brightness, the latter two sterol probes are clearly the better mimics of their natural counterparts; *i.e.*, ergosterol and cholesterol (for other sterol labeling techniques see [24,28]). Nevertheless, BODIPY-cholesterol probes with different orientation have been successfully used for studying sterol dynamics in the plasma membrane, revealing fast and unhindered sterol diffusion at the nanoscale [29,30].

Basic assumptions in all quantitative imaging studies designated to determine membrane probe distribution are that the emitted fluorescence signal is proportional to probe concentration and that the fluorescence signal remains stable during the image acquisition. Both assumptions have to be validated in each imaging application and for each fluorophore. Especially the latter assumption is often not valid, since in long-term observations, fluorescent probes become destructed by photobleaching. The emission of some fluorophores for a given probe concentration can depend on the local intracellular environment. For example, in cellular studies employing NBD- tagged probes, environmental factors need to be taken into account [31]. Benson *et al.* have shown by pixel-wise fitting of a mono-exponential decay model that photobleaching rates of NBD-cholesterol vary by more than one order of magnitude in cells [32]. Pagano *et al.* also observed—but did not quantify—heterogeneous photobleaching of NBD-phosphatidic acid (NBD-PA) and, after metabolic conversion, NBD-triacylglycerol [33]. Cholesterol-depletion accelerated photobleaching of C6-NBD-Cer in the Golgi apparatus of human skin fibroblasts [34]. In a pioneering study, Thomas Hirschfeld demonstrated almost 40 years ago that these effects of locally varying quantum yield can be overcome by measuring the time-integrated emission (TiEm) of a fluorophore until it is completely photobleached [35]. He showed that the TiEM is independent of the fluorescence quantum yield but also independent of the illumination intensity. This result has been used by some authors to correct for eventual probe self-quenching of NBD-tagged lipids before quantification of their organelle-associated fluorescence (self-quenching can lower the quantum yield and thereby the collected fluorescence signal) [11]. However, simple time-integration of the emitted fluorescence in living cells is not reliable, since cells contain locally varying autofluorescence which will also be integrated by that approach [36]. Here, we present a simple model for the photobleaching process and describe, how that can be used for ease analysis of photobleaching characteristics and discrimination of probe- from autofluorescence in cells. We show how the TiEm is derived from that model and used for correcting illumination shading of intracellular probes. In addition, we present a new implementation of pixel-wise calculation of TiEm in our recently developed plugin to ImageJ [37]. Finally, we extend the model to analyze multi- and non-exponential bleaching processes.

## 2. Results and Discussion

### 2.1. Limits of Fluorescence as Measure of Probe Concentration 

As pointed out in the previous section, one of the major goals of quantitative fluorescence microscopy is to determine the relative amount of tagged molecules in individual intracellular organelles for varying conditions and eventually over time. Since fluorescence of the probes is used as readout, one has to make sure that it is proportional to probe concentration. The detected fluorescence intensity at a given wavelength, λ, is given by [38], see Equation (1):

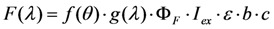
(1)


Here, 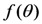 is a geometric factor related to the collection efficiency of the optical system with *θ* being the local convergence angle of the illumination/collecting beam at the sample plane (*i.e.*, fluorescence microscopy is a form of reflected light microscopy, where the objective plays also the role of the condenser). The wavelength-dependent quantum yield of the detector is 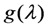, while 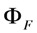 denotes the quantum yield of the fluorophore. The excitation intensity is *I*_ex_ and is given in W/cm^2^. Using the relation 1 W = 1 J/s, it follows that *I*_ex_, sometimes also named irradiance, is equivalently given in J/(s cm^2^). It can be also expressed as photon flux with units photons/(s cm^2^) by using the energy of illumination photons of a given wavelength, *i.e.*, 
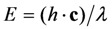
 with Planck’s constant as *h* = 6.626 × 10^−34^ J∙s and the speed of light as **c** = 300,000 km/s = 3 × 10^17^ nm/s [39]. This would give, for example, for a wavelength of *λ* = 320 nm, as used for excitation of DHE, *E* = 6.211 × 10^−19^ J per photon. Accordingly, an excitation energy of 1 W/cm^2^ = 1 J/(s∙cm^2^) corresponds to 1.61 × 10^18^ photons/(s∙cm^2^). Similarly, for blue photons with *λ* = 500 nm, as used for excitation of BChol, 1 W/cm^2^ corresponds to 2.515 × 10^18^ photons/(s∙cm^2^). In Equation (1), *ε* is the molar extinction coefficient (in M^−1^ cm^−1^), *b* is the optical path length (in cm) and *c*, is the probe concentration (in M). This equation, though central for our purpose, assumes fluorescence being linearly related to excitation energy. It is therefore strictly valid only for highly diluted solutions, in which fluorophores do not interact and for low irradiation, where fluorophores do not saturate. Thus, Equation (1) does not tell about the limitations of fluorescence detection for measurement of probe concentration, namely fluorescence quenching, saturation and photobleaching. To understand these processes, it is very instructive to start with some photokinetic considerations. 

The scheme shown in Equation (2) describes the simplest photocycle,

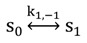
(2)
in which a fluorophore is either in the singlet ground state, *S*_0_, or, upon excitation with light of a suitable wavelength, resides in the first excited singlet state, *S*_1_. This gives the following two coupled differential Equations (3) and (4):

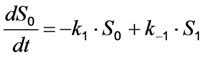
(3)

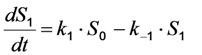
(4)


Here, the rate constant for absorption is 
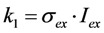
, with 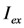
being the illumination intensity (as irradiance in W/cm^2^ or as photon flux in photons/(s∙cm^2^), see above, and 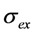 is the absorption cross-section of the fluorophore (in cm^2^ per molecule) for the given wavelength of excitation. The absorption cross-section is related to the extinction coefficient as 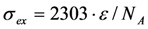, where *N*_A_ is Avogadro’s number (6.0221 × 10^23^ mol^−1^). Thus, *k*_1_ is for each molecule given in W = J/s, or equivalently, if the energy of illumination photons of a given wavelength is considered (as described above), in photons/s [39]. The measurable fluorescence lifetime, 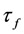, is the reciprocal of the rate constant *k*_−1_ and comprises radiative and non-radiative deexcitation. The solution of this system can be easily found by standard methods and reads for *S*_1_(*t*) for the initial conditions *S*_0_(*t =* 0) = *M* (the number of illuminated molecules) and *S*_1_(*t =* 0) = 0, see Equation (5):

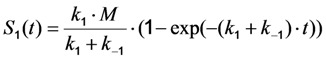
(5)


**Figure 1:**
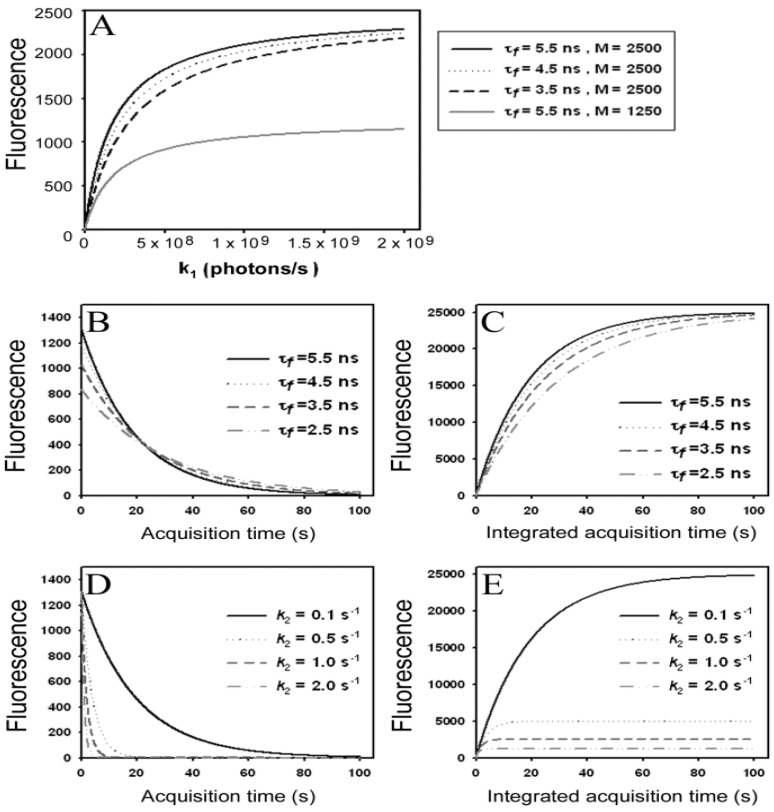
Simulation of fluorescence saturation, photobleaching and time-integrated emission using BChol as example. (**A**) fluorescence saturation was simulated from the time-independent part of Equation (5) as function of the rate constant for excitation, *k*_1_=*σ_ex_*·*I_ex_*. The illumination intensity, *I_ex_*, varied from 0 to 1 × 10^25^ photons/(s·cm^2^) corresponding to 0 to 2.5 × 10^5^ W/cm^2^, and 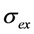, the extinction coefficient was set to the value of BChol (1.32842 × 10^−16^ cm^2^). The number of fluorophores is given by *M*, while the fluorescent lifetime is *τ_f_* = 1/*k*_−1_ (in ns); (**B**) simulation of photobleaching using Equation (10), as function of the fluorescence lifetime (values are indicated in the panel) for a fixed excitation rate constant of 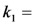 2 × 10^5^ photons/s and bleach rate constant 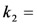 0.1 s^−1^, and *M* = 2500; (**C**) simulation of fluorescence as function of integrated acquisition time using Equation (14) and same parameters as in panel B. The influence of the intrinsic bleach rate constant, *k*_2_, on photobleaching kinetics (**D**) and integrated fluorescence (**E**) is simulated according to Equations (13) and (14), respectively, using a fluorescence lifetime of *τ_f_* = 5.5 ns, 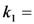 2 × 10^5^ photons/s and *M* = 2,500. See text for further explanations.

The measured fluorescence is proportional to the occupation of the excited state (*i.e.*, the collection efficiency of the detector and the radiative decay rate of the fluorophore enter as proportionality constants [35,40]). We will ignore these factors in the following and consider fluorescence synonymous with population of the excited state (see Figure 1).

It can be seen from Equation (5) that the solution for *S*_1_(*t*) consists of a time-dependent and independent part. The existence of the latter can be already inferred from inspection of Equations (3) and (4), since the corresponding matrix of coefficients is singular, which is characteristic for a closed kinetic system. When plotting the time-independent part of Equation (5), (*i.e.*, 
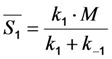
) as function of excitation intensity, one finds a saturation curve, where for increasing intensity, *I*_ex_, the plot deviates from a linear relationship (Figure 1A). Here, we used the mean fluorescence lifetime of BChol, a recently introduced fluorescent analog of cholesterol, as measured in model membranes [41] and in cells (*i.e.*, *τ_f_* ~5.5 ns) [26]. Accordingly, one needs to set the excitation energy and acquisition time as short as possible for obtaining good signal-to-noise ratios with maximal detector sensitivity.

Another complicating factor is eventual self-interaction of membrane probes, so called self-quenching. This has been reported for various lipids tagged with nitrobenxoxadiazole (NBD)- and BODIPY-dyes as well as for rhodamine-based membrane probes [42,43,44]. Alternatively, fluorescence of lipid probes can be quenched by various mechanisms due to the presence of intracellular pigments or proteins. If the quenching takes place in the ground state due to formation of dimers or larger aggregates, less fluorophores are available for excitation thereby lowering the overall observed probe fluorescence (so-called ‘static quenching’). Evidence for static quenching of BChol has been provided above 3 mol% in POPC membranes based on: (i) concentration-independence of probe fluorescence for high BChol concentration and (ii) by occurrence of a new absorption band above 3 mol % being characteristic for excitons (ground state dimers) [26]. Alternatively, quenching can take place by self-interaction of the excited fluorophores, which can lower the measured fluorescence lifetime [44]. Collisions with some quenchers during the excited state lifetime of the fluorophore, so-called ‘dynamic quenching’, can also take place and will shorten the measured fluorescence lifetime, since an additional relaxation channel has been introduced by the quencher.

According to the model shown in Equations (2)–(5), static and dynamic quenching result in very different predictions for the fluorescence saturation curve (Figure 1A): fluorescence saturation in the presence of static quenching due to complex formation between fluorophore and quencher takes place at the same excitation intensities than in the absence of quencher but with a lowered plateau due to the lower number of fluorophores being available for excitation (Figure 1C, grey, straight curve with *M* = 1,250 molecules). Note, that the initial amount of fluorophores, *M*, is arbitrarily chosen and just serves illustrative purposes. An equivalent treatment would be to normalize to the total number of fluorophores and express the excited state population as fraction or in percentage (see below and Supporting Information). Dynamic quenching does not alter the fluorescence plateau but shifts the saturation point to higher excitation energies due to lowered fluorescence lifetime (Figure 1A, black straight and dashed curves with *M* = 2,500 molecules and *τ_f_* = 3.5–5.5 ns). Accordingly, acquiring images of a fluorescent probe at different excitation energies could be an alternative method to fluorescence lifetime imaging for determining mechanisms underlying eventual fluorophore quenching. We tested this idea for BChol in living cells on a confocal microscope but were limited by the relatively narrow linear range of our photomultiplier tube detector (not shown). In fact, optical saturation measurements are normally performed in solution and use more elaborative equipment including pulsed laser excitation and CCD cameras as detectors [45]. In addition, in the complex intracellular environment many other factors contribute to the overall fluorescence signal (e.g., spatially varying intersystem crossing to triplet states, ground state heterogeneity due to probe partitioning into media with varying properties, photobleaching or varying availability of oxygen, which is a known singlet and triplet state quencher). The time-integral of Equation (5) goes for *n* → ∞, with *n* being the number of acquisitions against infinity. Accordingly, for a non-bleaching fluorophore an infinite number of photons can be collected.

### 2.2. A Simple Model for Photobleaching and Time-Integrated Emission

Real fluorophores become destructed by intense and/or prolonged excitation and thereby irreversibly removed from the photocycle. Photobleaching can be included in the model shown in Equations (2)–(5) by adding an irreversible reaction step to the excited state giving Equation (6):

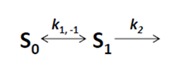
(6)
where *k*_2_ is the intrinsic bleach rate constant. We get Equations (7) and (8):

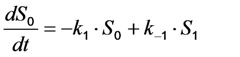
(7)

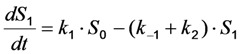
(8)


For that model, the coefficient matrix has a non-vanishing determinant being characteristic for an open kinetic system. It can be solved analytically [36], but for further extension, we will introduce a simplification here. Suppose, that the photocycle of excitation and radiative or non-radiative relaxation is much faster than photobleaching (*i.e.*, *k*_1_, *k*_−1_ >> *k*_2_), we can use a rapid equilibrium approach (REA), as recently described [46], see Equation (9):

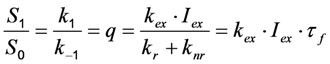
(9)
and therefore Equation (10):

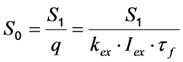
(10)


Thus, rapid equilibration of the photocycle after a very short time span gives for continuous excitation the amount of fluorophore in both states as Equation (11):

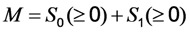
(11)
such that Equation (12):

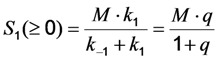
(12)


This leads to one differential equation for the excited state, whose population is proportional to the emittable fluorescence according to Equation (13):

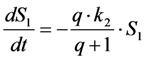
(13)


Using the initial conditions given in Equation (12), we obtain Equation (14):

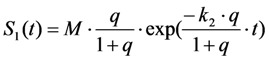
(14)


Please note, that this system does not contain a time-independent part, but the amplitude of the decay in Equation (14) equals the time-independent part of the model without bleaching (see Equation (12)). According to Equation (14), the measured or observable bleach rate constant is a function of the intrinsic bleach rate constant, *k*_2_, the excitation intensity, *I*_ex_, and the fluorescence lifetime, *τ_f_*. Dynamic quenching lowers the fluorescence lifetime such that the initial intensity 
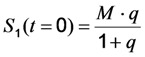
 as well as the observed photobleaching rate constant 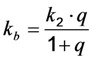 of a fluorophore will be lowered in the presence of a collisional quencher due to an effective decrease of *q* (Figure 1B). 

Note again, that we ignored the proportionality constants between the population of molecules in the excited state, *S*_1_, and measured fluorescence signal for the sake of brevity. Taking these constants into account will not affect the results of our analysis. The initial intensity, 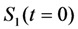, and phenomenological photobleaching rate constant, 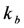, show saturation behavior for high values of *q* (in photons/molecule), which can, for example, simulate increasing illumination intensity (not shown, but see [46]). Such saturation of fluorescence and bleach rate constant as function of illumination has been measured for the BODIPY and rhodamine fluorophore [47,48]. A static quencher will only lower the number of fluorophores participating in the photocycle, *i.e.*, *M* in the model, but not the photobleaching kinetics (not shown). In general, any differences in fluorescence lifetime caused by locally varying fluorescence quantum yield in the cellular environment should be ‘detectable’ as a small change in photobleaching rate constant, as illustrated in Figure 1B. This effect has been used in the so-called donor photobleaching method for detecting Förster resonance energy transfer between fluorophore couples [49,50,51]. Since image acquisition requires integration of the fluorescence signal over some time period, varying the fluorescence lifetime for a given intrinsic photobleaching rate constant (e.g., *k*_2_ = 0.1 s^−1^) could affect the detected intensity, even in the first acquired frame. To test this in more detail, we calculate the time integral, *F*, for Equation (14) giving Equation (15):

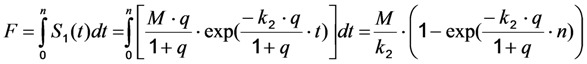
(15)


As shown in Figure 1C, the time integral shows a plateau for very long acquisition times because all fluorophores became photobleached (*i.e.*, after more than 100 s in this example). For a given total acquisition time, (<<100 s) the measured intensity will vary as function of the fluorescence lifetime (as simulated by varying *q*), while the total time integral is independent of fluorescence lifetime and quantum yield in accordance with earlier predictions [35]. Thus, Equation (15) reduces to *M*/*k*_2_ for *n* → ∞ and *F* becomes the TiEm. Let’s denote this as Equation (16):

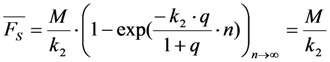
(16)


Thus, any relative increase of the intrinsic bleach rate constant, *k*_2_, causes an equally large decrease in 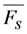, the TiEm from the excited singlet state. Reasons for variation of the intrinsic bleach rate constant can be manifold. For example, in cell membranes *k*_2_ could vary due to different availability of molecular oxygen for photooxidation. Differences in detected TiEm due to variation of the intrinsic photobleaching rate constant, *k*_2_, can thus become very large, since for lower values of *k*_2_, it requires longer illumination and more photocycles to totally bleach the pool of available fluorophores (Figure 1E). In other words, the total fluorescence being collectable from a given number of fluorophores before the whole population is bleached depends on intrinsic properties of the probe, like its intrinsic bleach rate constant, *k*_2_, but not on the fluorescence lifetime, *τ_f_*, as first shown in an elegant study by Hirschfeld [35], and reconciled here (compare Figure 1C,E). 

Interestingly, Hirschfeld did not take fluorescence saturation into account in his model but still arrived at an expression equivalent to Equation (16), above. Literally, he received an expression, using our nomenclature, of 
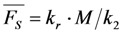
, since he included the dye-specific radiative lifetime as proportionality constant (see Equation (9) in [35]). Finally, the simple model of Equations (14) and (15) allows for deriving the TiEm solely from the acquired bleach curve. Let’s assume one performs a non-linear regression of a mono-exponential decay function of the form 
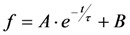
 to a measured bleach curve recorded for a fluorophore in cells. Here, the bleach time constant determined by the fit is *τ* = 1/*k_b_*, and *B* is a measure of the background autofluorescence (assumed to bleach very slowly, see [36,52]). The decay amplitude, *A*, again, can be associated with the initial intensity of Equation (14), *i.e.*, 
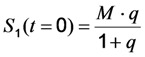
. Direct integration of this mono-exponential decay function results in a linear term B·*t* in the time integral, such that the estimated TiEm would be compromised by autofluorescence (see Supplemental Information and discussion in [36]). With the model in Equation (14) and by having 
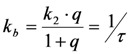
, we get the TiEm of Equation (16) simply as the product of *A* and *τ*, thereby automatically correcting for autofluorescence!

### 2.3. Validation of the Model Using Synthetic Fluorescence Images

**Figure 2:**
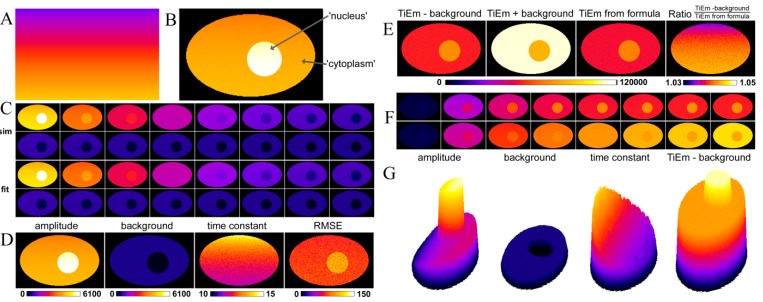
Analysis of synthetic fluorescence images with illumination shading using PixBleach. Synthetic images were simulated using the Java-based Macro language of ImageJ, using the photobleaching model on a pixel-by-pixel, Equation (17), as described in the text. Spatially varying illumination intensity simulated by a ramp-like *q*-map (**A**) was used as input for the model at each pixel position with additional random variation of the intrinsic bleach rate constant, *k*_2_, according to Equation (18). The first frame of this simulation with *M*_1_ = 5000 and *M*_2_ = 7000 being constant throughout the cytoplasm and nuclear compartment, respectively, is shown in panel (**B**); (**C**) selected frames of the simulated bleach stack (**C**, upper two rows) and the reconstructed data from pixel-wise fitting of a mono-exponential decay to the synthetic image set (**C**, lower two rows); Panel (**D**) shows fitting results also with a FIRE LUT, from left to right (‘amplitude’, background’, ‘time constant’ and ‘RMSE’); (**E**) various forms of the time-integrated emission (TiEM); from left to right, calculated as summed pixel intensity in PixBleach, either without including background into the calculation (‘TiEm-background’) or with the estimated background included (‘TiEm + background’) and as product of the regression-derived amplitude and time constant (‘TiEm from formula’). The most right panel in E show the ration of ‘TiEm-background’ and ‘TiEm from formula’ in the indicated range (1.03 in dark blue to 1.05 in yellow/white). G, surface plots of the indicated parameter maps. See text for further explanations.

To mimic a field of uneven illumination, we generated an image with gradual change of *q*-values on a line-by-line basis from *q*(*x*, *y*) = 8.0 photons/molecule at the bottom to *q*(*x*, *y*) = 2.0 photons/molecule at the top (Figure 2A). This simulates spatially varying illumination intensity. A synthetic cell image was generated simulating the distribution of a substance between the nucleus with constant amount of *M* = 7,000 molecules on a background of 100 intensity units and the cytoplasm, where *M* = 5,000 molecules on a background of 600 intensity units (Figure 2B). The background term, *B*, resembles cellular autofluorescence in the respective compartment (photophysics of background is not considered). Using the *q*-map of Figure 2A, bleaching fluorescence was simulated by solving Equation (14) on a pixel-by-pixel basis, as defined in Equations (17) and (18), below. To mimic local variations in the intrinsic bleach rate constant, as it might occur in a real cell, we set:

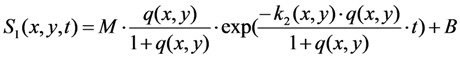
(17)
where *k*_2_ (*x*, *y*) was given by:

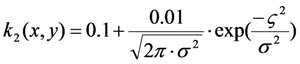
(18)


The additive Gauss-term in Equation (18) with amplitude equal to 0.01, mean equal to zero and variance *σ*^2^ = 1 was generated in ImageJ by implementing the Box-Muller method, as described [53,54]. That is, for each pixel a new normal-distributed random number was generated initially, thereby changing the bleach rate constant, *k*_2_ (*x*, *y*), and thereby the bleaching behavior slightly from pixel to pixel. As result, we get spatially varying intensity (*i.e.*, amplitude) in the first frame (due to the varying *q*-term; Figure 2B) and heterogeneous bleaching kinetics (due to both, the varying *q*- and *k*_2_ values; Figure 2C, upper two rows). Pixel-wise fitting of a mono-exponential bleaching model implemented in PixBleach gave a perfect reconstruction of the data (Figure 2C, lower two rows). Moreover, the ‘illumination shading’ simulated by spatially varying *q*-maps was found in the derived amplitude- and time-constant-maps, in full agreement with Equations (14), (17) and (18) (Figure 2D, most left and next right panel).

The background term showed no spatial trend and was correctly determined in the nucleus and cytoplasm with values of *B* = 103.4 ± 7.4 and *B* = 602 ± 5.3, respectively (Figure 2D, next left panel). The quality of fit, judged by the root mean squared error (‘RMSE’) was good and without notable shading effect (Figure 2D, most right panel). The fit using the latest version of PixBleach provides the integrated signal as summed intensity of the reconstructed stack, either without or with background (Figure 2E; ‘TiEm –background’ and ‘TiEm + background’), respectively. We also determined the TiEm as product of amplitude- and time constant map (Figure 2E, next right panel named ‘TiEm from formula’) and calculated the ratio of ‘TiEm-background’ and ‘TiEm from formula’ (Figure 2E most right panel). The latter shows that both ways of calculating the TiEm give identical results with ~73,000 and ~52,000 intensity units in the nucleus and cytoplasm, respectively. Thus, the TiEm calculated in both ways corrects for the different values of the background term in both compartments. Integration of the fluorescence signal including the background terms gives deviating results. PixBleach also provides the integrated signal as function of acquisition number in an image stack as spatial implementation of Equation (15) (Figure 2F). This, again, clearly shows that integration in presence of the background term overestimates the true TiEm (compare Figure 2F lower and upper row). Surface plots generated from the data for the amplitude-map, the background estimated by the fit, the time-constant-map and the ‘TiEm-background’ (Figure 2G) support these conclusions.

### 2.4. Validation of the Model Using Live-Cell Images of Dehydroergosterol-Labeled Fibroblasts

**Figure 3:**
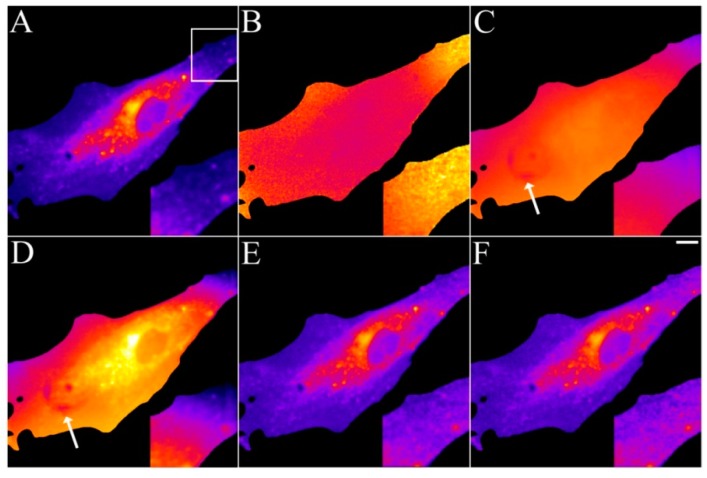
Removal of background and illumination shading in DHE-labeled fibroblasts. Human fibroblasts were labelled with DHE/MCD for 3 min, followed by a chase in buffer medium for 1 h at 37 °C. Bleach stacks were acquired on a UV-sensitive wide field microscope and fitted on a pixel-by-pixel basis to a mono-exponential decay function in PixBleach. The fit provides the amplitude-map (**A**), the time constant map (**B**) and the background map. The TiEm calculated including the estimated background reveals image shading and some dark pixels stemming from the camera (arrows). The TiEm without background was either calculated from the summed pixel intensities (**E**) or as product of amplitude and time constant (**F**). Insets corresponding to the box in panel (**A**) illustrate the correction for illumination shading in panel (**E**,**F)**. Bar, 10 μm.

Having tested the model on synthetic images, we aimed for an experimental realization and test with real image sequences acquired on a fluorescence microscope. Earlier studies by Benson *et al.* found that NBD-tagged cholesterol had very heterogeneous photobleaching kinetics in different areas of cells [32]. The authors suggested that this is due to local self-quenching as well as different availability of molecular oxygen. They concluded that quantitative studies of intracellular sterol distribution using fluorescence microscopy should be interpreted with caution. We have previously shown that DHE bleaches homogeneously in the plasma membrane, ERC, vesicles and the cytoplasm and also found that its fluorescence is proportional to mole fraction in membranes up to 45 mol % of DHE [52,55]. Thus, we can safely state that the imaging studies using DHE performed so far do not suffer from the problems Benson *et al.* observed for NBD-cholesterol. This is somehow not surprising, since DHE shows relatively little environmental sensitivity of its fluorescence lifetime, while the opposite is true for NBD-tagged lipids [56,57]. Thus, imaging of DHE with spatially varying illumination should allow us testing the model on real cell images. For that purpose, human skin fibroblast were labelled with DHE from a sterol-cyclodextrin complex and imaged on a UV-optimized wide field microscope, as described [52]. Spatially varying illumination was observed as shading in case of slight misalignment of the bulb of the mercury arc lamp. In addition, the used camera (a UV-sensitive back-thinned CCD from Hamamatsu, model Orca 2.0) generated some dark pixels in the image field, which were independently resolved by taking images with closed shutters (not shown). Stacks of DHE-labeled fibroblasts were acquired, and the intensity loss due to photobleaching was analysed using PixBleach, as described above for the synthetic images. The amplitude-map nicely reveals the DHE distribution but showed some slightly lowered intensity at the cell edge in the otherwise homogeneously stained plasma membrane (Figure 3A, zoomed box). 

The time-constant-map confirmed that photobleaching was slower in the upper right corner than in other areas of the cell (Figure 3B). The background map was rather inhomogeneous and revealed the dark pixels generated by the detector, in the central area of the cell (arrow in Figure 3C). The time-integrated signal in the presence of background enhanced the background inhomogeneity including the dark pixels and the shading across the field (Figure 3D). The TiEm calculated from either the integrated signal excluding the background term (Figure 3E) or from the product of amplitude- and time-constant-map (Figure 3F) corrected for both artefacts. Since we verified in independent measurements in liposomes, that DHE’s intrinsic bleach rate constant is independent of probe concentration [58] and other factors (not shown) in membranes, the TiEm should represent a true measure of DHE distribution in cells. The TiEm differs also from the amplitude-map in areas where illumination shading was pronounced (compare insets in Figure 3A and Figure 3E,F). The results clearly confirm the value of pixel-wise calculation of TiEm for excluding background, autofluorescence and illumination shading. In case of spatially invariant intrinsic bleach rate constant, *k*_2_, the TiEm is a reliable measure of probe distribution.

### 2.5. Analysis of Photobleaching and Time-Integrated Emission in the Presence of a Triplet State

Photobleaching takes often place from the triplet state after intersystem crossing in the excited fluorophore [59,60]. This can be easily included in the model as follows. By setting the intersystem crossing rate constant as *k*_3_, the triplet state can be included giving Equation (19):

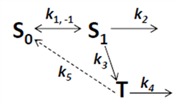
(19)


Here, the intrinsic bleach rate constant from the triplet state, *T*, is *k*_4_, while the transition from the triplet to the singlet ground state is described by *k*_5 _(both rate constants are in s^−1^). The differential equation system describing the system shown in Equation (19) including the REA between *S*_0_ and *S*_1_ is Equations (20) and (21):

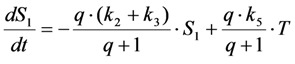
(20)

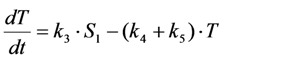
(21)


This system can be written in matrix form as Equation (22):

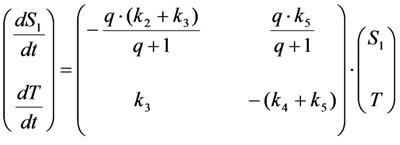
(22)


The eigenvalues of the coefficient (Jacobian) matrix, **A**, in Equation (22) read as Equations (23) and (24):

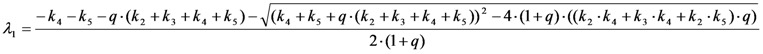
(23)

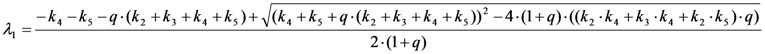
(24)


This can be also written as Equations (25)–(27):

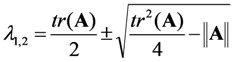
(25)
with:

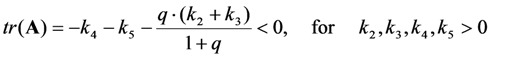
(26)

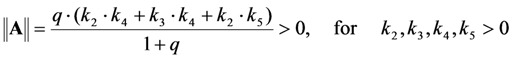
(27)


Since we also have 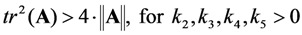, it follows directly from the Rough-Hurwitz criterion that 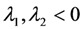 and 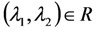. The corresponding eigenvectors and thereby the solution of Equations (20) and (21) can be also calculated but give formidably complex expressions (not shown). The presence of two different eigenvalues results in a bi-exponential bleaching decay with dependencies on all involved rate constants. Accordingly, also the interpretation of TiEm in the presence of a triplet state, 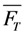, will be less straightforward than in the case of bleaching from a singlet state only. The only remaining feature is that 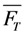 remains, as 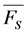, proportional to the number of fluorophores, *M*. Accordingly, in both cases, presence of static fluorophore quenching by complex formation would lower the TiEm proportionally and independent of the actual bleaching kinetics. Bi-exponential photobleaching due to photoreaction from the triplet state has been observed for fluorescein [60,61]. 

In many experimentally relevant situations we can simplify the differential equation system originating from Equation (19) even further. Fluorescence lifetimes of the excited singlet state are in the nanosecond range, while intersystem crossing and relaxation from the first triplet state (radiative as phosphorescence or non-radiative) typically take place in micro- to milliseconds for many dyes [59]. Photobleaching, however, occurs typically on a time scale of seconds, which allows us to extend the REA to all three states of the system in Equation (19). Thus, we assume that all states come to equilibrium long before significant photobleaching from either the excited singlet or the triplet state takes place. This gives Equations (28)–(30):

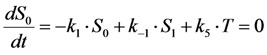
(28)

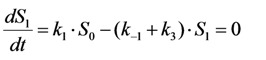
(29)

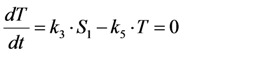
(30)


From Equations (29) and (30) we get Equations (31) and (32):

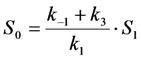
(31)

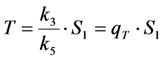
(32)


Now, using this in the model including bleaching (Equation (19), we get Equation (33):

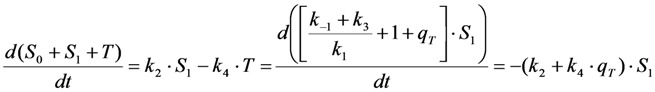
(33)


After some elementary rearrangements, one gets the differential equation describing fluorescence in the presence of photobleaching from *S*_1_ and *T* as Equation (34):

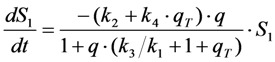
(34)


Here, *q* is defined as above as
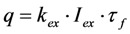
in photons/molecule. Using the initial condition 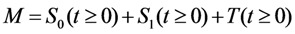 and Equations (31)–(33), above, we obtain Equation (35):

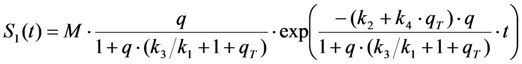
(35)


The integrated intensity becomes Equation (36):

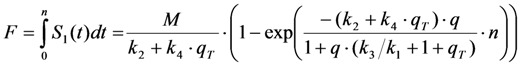
(36)


**Figure 4:**
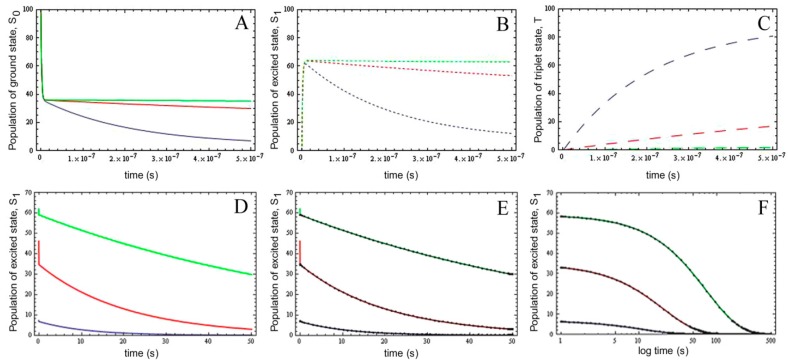
Validation of the extended photobleaching model in the presence of varying triplet yield. The complete kinetic model shown in Equation (19) was solved numerically using a Runge-Kutta scheme implemented in Mathematica 7.0 (Wolfram research Inc., Champaign, IL, USA). (**A**–**C**) occupation of the singlet ground state, S_0_ (**A**, straight lines), the singlet excited state, S_1_ (**B**, short dashed lines) and the first triplet state, T (**C**, long dashed lines) for different intersystem crossing rate constants ranging from *k*_3_ = 6.6 × 10^3^ s^−1^ (green lines), over *k*_3_ = 6.6 × 10^4^ s^−1^ (red lines) to *k*_3_ = 6.6 × 10^5^ s^−1^ (blue lines) on the short time scale (*i.e.*, ns–μs). D-F, dynamics of the excited singlet state on the long time scale for the same conditions; In (**D**), the decay from the excited singlet state according to the full numerical solution is shown. The analytical approximate solution based on the REA (Equation (35) closely matches the long-term dynamics of S_1_; (**E**, black lines and symbols show the analytical solution on top of the numerical simulation (colored curves)); Panel (**F**) is like (**E**) but with a logarithmic time axis to show the coincidence of numerical and analytical model up to 500 s.

Importantly; the photobleaching kinetics as well as the time integral follows again a mono-exponential function; as in case for photobleaching from the excited singlet state; only (see above). To validate the extended model; we performed numerical simulations of the full model (see Equations (19) and (28)–(30)) using parameter values derived for fluorescein isothiocyanate and for rhodamine dyes [59,61]. That is; we used an excitation rate constant of *k*_ex_ = 3.8 × 10^8^ photons/s; a decay rate constant from the excited singlet state of *k*_−1_ = 2.134 × 10^8^ s^−1^; an intersystem crossing rate constant between *k*_3_ = 6.6 × 10^3^ s^−1^ and *k*_3_ = 6.6 × 10^5^ s^−1^ and a decay rate constant from the triplet state of *k*_5_ = 5 × 10^4^ s^−1^ (Figure 4A–C). 

In addition, we chose an intrinsic bleach rate constant from the excited singlet state, of *k*_2_ = 0.01 s^−1^ and from the excited triplet state of *k*_4_ = 0.1 s^−1^, respectively. These values matched the observed bleaching kinetics of fluorescein [61,62]. By varying the intersystem crossing rate constant, we aimed for assessing the effect of varying triplet yields on the probe photophysics and photobleaching probability. There are several time scales visible in the photocycle: (I) in the ns time range, the excited state becomes populated, and for low triplet yields, an equilibration between singlet ground and excited state can be observed (Figure 4A,B, green lines); (II) Depopulation of S_0_ and S_1_ is observed in tens of ns up to μs as a consequence of build-up of the triplet state, which is most pronounced for large intersystem crossing rate constants (*i.e.*, *k*_3_ = 6.6 × 10^5^ s^−1^; blue lines in Figure 4A–C); (III) On a much slower time scale, photobleaching from the excited singlet and triplet state further depopulate all molecular states (Figure 4D–F, only shown for S_1_ with the same parameter settings). Importantly, the analytical solution to Equation (19) using the REA (see Equation (35)) exactly matches the slowest dynamics (*i.e.*, (III) above), but cannot describe the initial population of the electronic states (compare colored lines and black lines and symbols in Figure 4E).

The analytical simplified description holds for time scale III until the fluorophore becomes totally photobleached, as indicated on a logarithmic time scale (Figure 4F). To explore further under which conditions, the analytical model based on the REA might fail and for the purpose of illustration, we varied next in parallel the intersystem crossing rate constants from *k*_3_ = 6.6 × 10^2^ s^−1^ to *k*_3_ = 6.6 s^−1^ and the decay rate constant from the triplet state from *k*_5_ = 50 s^−1^ to *k*_5_ = 0.5 s^−1^, thereby keeping the triplet yield unchanged (all other parameters were as described in Figure 4; see Supporting Information). We found increasing deviation of the analytical model from the complete numerical simulation of Equations (28)–(30) (see Supporting figure for triplet yields of 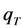 = 660/50 = 13.2, cyan line; 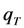 = 66/5 = 13.2, pink line and 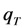 = 6.6/0.5 = 13.2, blue line). In fact, when the intersystem crossing rate constant is only 66fold higher than the bleach rate constant from the triplet state, the analytical model misses more than the first sec of observed photobleaching (see Supporting figure, blue line). In other words, when significant bleaching takes place BEFORE the equilibration of the electronic states on the first two time scales (see above), our model cannot describe the photobleaching dynamics. Thus, fluorophores bleaching preferentially from the triplet state but having very low spin-orbit coupling in a given environment cannot be studied with our photobleaching model. The model outlined in Equations (20) to (27), in which the triplet state dynamics is explicitly taken into account would account for this situation. 

**Figure 5:**
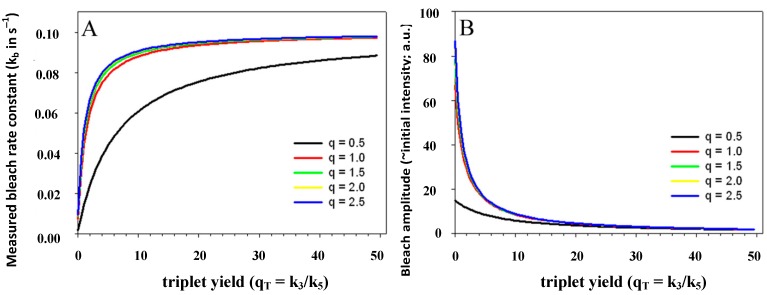
Impact of varying triplet yields on measured photobleaching parameters. Plot of the experimentally accessible bleach rate constant (**A**) and bleach amplitude (**B**) of the extended analytical model (Equation (35) as function of the triplet yield, 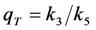, and equilibrium constant of the photocycle between the two singlet states,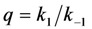. The triplet yield was varied between zero (no triplet occupation) and fifty (high triplet occupation) on the abscissa. Other parameters were as for Figure 4, *i.e.*, an intrinsic bleach rate constant from the excited singlet state, of *k*_2_ = 0.01 s^−1^ and from the excited triplet state of *k*_4_ = 0.1 s^−1^. Variation of the equilibrium constant of the photocycle between the two singlet states, *q*, is as indicated in the figure.

The triplet yield given by the ratio of intersystem crossing rate constant and decay rate constant from the triplet state (*i.e.*, 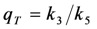) is known to vary significantly not only between different fluorophores, but also in different environments. This has been shown, for example, for fluorescein in water *versus* ethanol [59]. As shown in Figure 5 in a simulation using the analytical bleaching model of Equation (35), the triplet yield has a profound effect on the bleaching amplitude, 
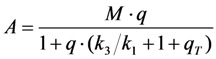
, and on the empirical bleaching rate constant of the mono-exponential decay, 
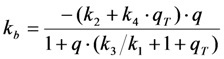
. Both experimentally easily accessible parameters depend hyperbolically on the illumination intensity, as derived for the case of bleaching from *S*_1_. In other words, both parameters show saturation for high *I*_ex_ also in case of presence of a triplet state. This has been also found in experimental studies of single dye molecules embedded in a polymer matrix [48]. In addition, the bleach rate constant increases in a hyperbolic manner with growing triplet yield, an effect which is most pronounced for high illumination intensities mimicking fluorophore saturation (Figure 5A). In addition, increasing bleaching from the excited singlet state (*i.e.*, increasing *k*_2_) affects the measurable bleach rate constant only for low triplet yields (not shown). The bleaching amplitude is inverse proportional to the triplet yield (Figure 5B). 

With our analytical approach, the TiEm again becomes independent of *q* and thereby independent of fluorescence lifetime and illumination intensity, as we get for *n* → ∞ the TiEm with triplet as Equation (37):

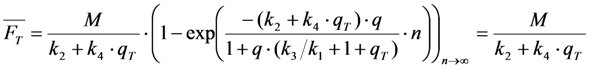
(37)


Thus, in the presence of triplet bleaching, any relative increase of the intrinsic singlet bleach rate constant, *k*_2_, and/or the intrinsic triplet bleach rate constant , *k*_4_, weighted by the equilibrium constant of the triplet state, 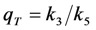, lower the attainable TiEm, 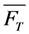. Variation in either the intersystem crossing rate constant or the relaxation rate constant from the triplet to the singlet ground state can therefore have profound impacts on the overall bleaching kinetics, especially if the bleach rate constant from the triplet state is significantly higher than that from the excited singlet state (*i.e.*, if *k*_4_ >> *k*_2_). Both intrinsic bleach rate constants, as well as the triplet yield will vary in different regions of the cell. Also, the TiEm with triplet bleaching is, as shown for the singlet state, conveniently given as product of amplitude- and time-constant-map from a mono-exponential fit to experimental data.

### 2.6. Multi-Exponential Photobleaching of Fluorescent Probes in Living Cells 

The model presented so far allows us to give a physical interpretation of mono-exponential bleaching kinetics in case of bleaching from an excited singlet or triplet state. Combination of both cases might occur in cells, if, for example, fluorescent probes experience different local environments (e.g., one probe population, in which the triplet state is quenched by oxygen, leading to Equation (14) and one, in which significant bleaching occurs also from the triplet state, Equation (35)). Let’s call the first population for *M*_1_ and the second *M*_2_, one would get for the photobleaching kinetics in case of identical fluorescence lifetime of both states Equation (38):

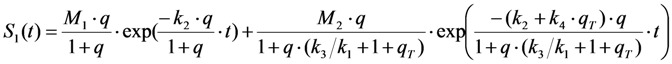
(38)


The TiEm will be the sum of 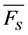 and 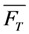, given by Equations (16) and (37), respectively. It is also possible, that a fluorophore has several distinct lifetimes in different cellular microenvironments. For example, NBD-tagged PC and SM species as well as BChol have been shown to possess bi-exponential fluorescence lifetime decays in living cells [26,56]. Apart from the possibility of more complex photophysics leading to multi-exponential photobleaching as discussed in Section 4, this could be a consequence of different protonation states in the excited state or due to two dye populations with varying quenching sensitivity [63]. Assuming photobleaching from singlet and triplet state (Equation (35)) but now with two different fluorescence lifetimes, this will lead to Equation (39):

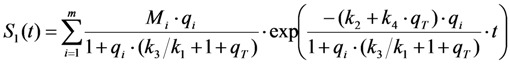
(39)
with *m* = 2. Similarly, multi-exponential lifetime decays with *m* > 2 can enter the model. The TiEm will be given by Equation (40):

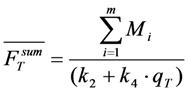
(40)


These different populations can co-exist in regions smaller than the resolution limit (e.g., microdomains) and thereby appear as bi-exponential bleaching in each pixel position. For example, a pixel size of 0.1 × 0.1 μm covers an area of 10,000 nm^2^ resembling a plasma membrane patch of about 12,000 lipids, and 300 proteins. In this mesoscopic region, many important membrane phenomena can contribute to spatially varying fluorescence properties of membrane probes. For example, nanodomain formation or surface invagination could result in varying bilayer packing. This, in turn, could cause different vibrational relaxation of the excited fluorophore affecting its lifetime or could result in locally differing availability of oxygen for quenching or bleaching processes. Similarly, slight variation in refractive index of the membrane-embedding solution (e.g., the cytoplasm) can affect the measured fluorescence lifetime, as shown for diphenylhexatriene and certain dicarbocyanine dyes [64,65]. 

As a consequence, the fluorescence of a probe acquired in the first frame as well as the photobleaching kinetics would be affected as well. Resolving multi-exponential photobleaching kinetics can thereby provide a glimpse of superresolution, *i.e.*, information about the probe microenvironment below the diffraction limit, similar as pixel-wise analysis of fluorescence lifetimes of intracellular probe molecules [35,56]. Empirical pixel-wise fitting is implemented in PixBleach for a bi-exponential fluorescence loss as 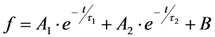 [36]. 

Using this expression, we can identify the sum of both empirical fitting amplitudes weighted with their respective bleach time constants again with the TiEm. For example, in case of a singlet and triplet population (Equation (38), we get Equation (41):

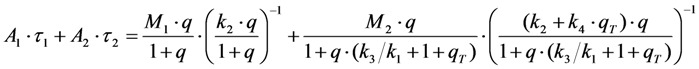
(41)
which is equivalent to Equation (42):

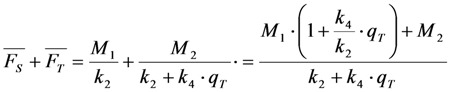
(42)


In case of two populations with identical singlet and triplet bleaching but two different fluorescence lifetimes (Equation (39)), we get Equation (40); *i.e.* Equation (43):

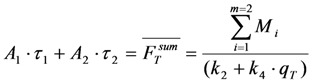
(43)


Importantly, in both cases, the TiEm remains independent of fluorescence lifetime and illumination intensity, *i.e.*, independent of *q*, and is given by the summed product of fractional amplitude and associated time constant.

**Figure 6:**
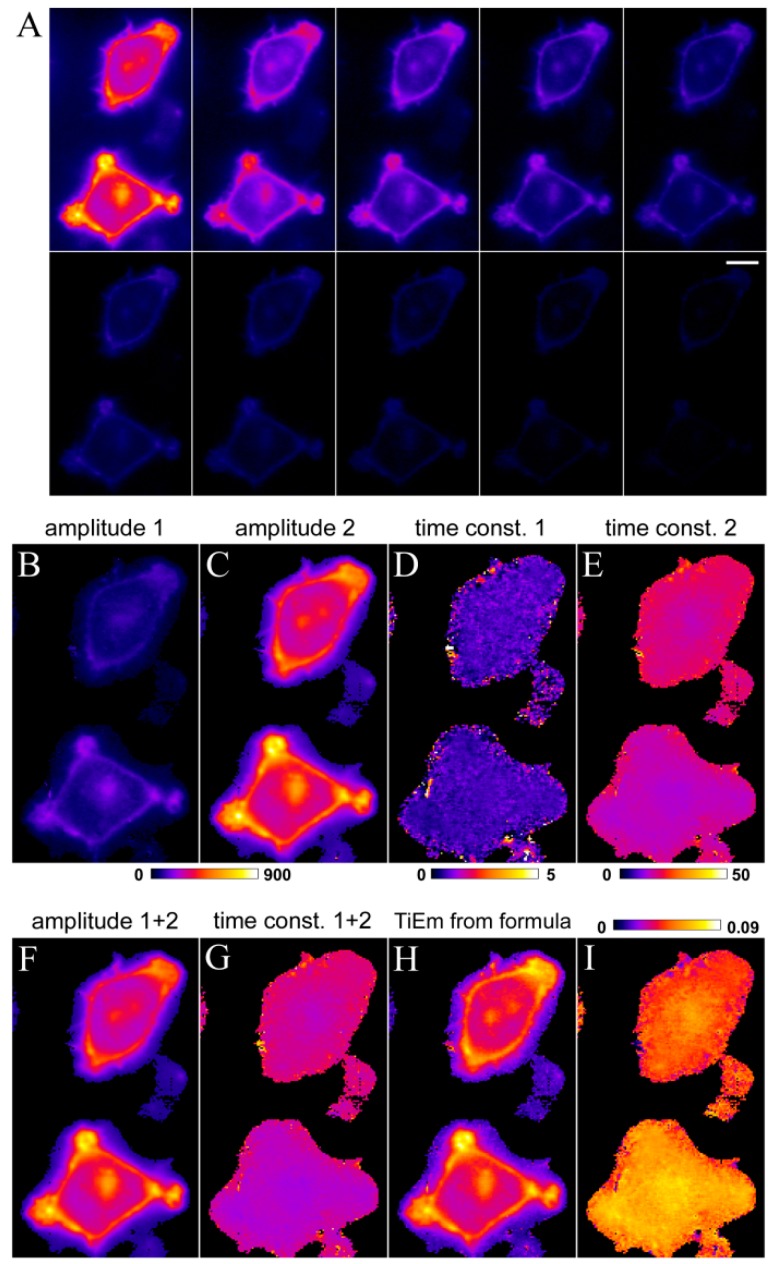
Correction for illumination shading in HeLa cells labelled with C6-NBD-SM. HeLa cells were labelled with C6-NBD-SM and imaged on a wide field microscope as described in Materials and Methods. Bleach stacks (example frames shown in **A**) were fitted on a pixel-by-pixel basis to a bi-exponential decay function in PixBleach. The fit provides two amplitude maps (**B**, **C**), the time constant maps (**D**, **E**) and the background map (not shown). The sum of both amplitudes and both time constant maps are shown in (**F**, **G**), respectively. TiEm calculated as product of amplitude and time constant (**H**) corrects for intensity differences between both cells due to spatially varying irradiance; (**I**) ratio of summed amplitudes and TiEm (*i.e.*, panel **F**, **H**) in the indicated range (0.0 in dark blue to 0.09 in yellow/white). Bar, 5 μm.

To validate the model on cellular images, we labeled HeLa cells with the sphingolipid analoge C6-NBD-SM and acquired bleach stacks on a wide field microscope (Figure 6A). 

Photobleaching of fluorescence of C6-NBD-SM was best described by a bi-exponential decay model providing two decay amplitued (Figure 6B,C) and two time constants (Figure 6D,E). The upper cell has less initial intensity (as seen in the sum of both amplitudes; Figure 6F) and bleaches slower (as seen in the sum of both time constants; Figure 6G) than the lower cell in the field. This points to some illumination shading with lower illumination intensity, *I*_ex_, in the upper half of the image, resulting in lower *q*-values. Clearly, the TiEm calculated by using Equation (41) or Equation (43); left hand side, corrects for this artefact and provides more equal cellular intensities of C6-NBD-SM (Figure 6H). This is true independent of which of the two photophysical mechanisms suggested above underlie the observed bi-exponential photobleaching kinetics. The ratio of summed amplitude and TiEm reveals the correction of a fluorescence intensity gradient given by uneven illumination (Figure 6I). Together, these results illustrate the use of TiEm for correction of illumination shading in case of multi-exponential bleach processes and verify the correctness of our model predictions.

### 2.7. Extension of the Photobleaching Model to a Random Distribution of Rate Constants 

The cellular microenvironment is very heterogeneous such that the ‘well-stirred assumption’ of classical chemical kinetics might fail. This could also apply to photobleaching of intracellular probes which eventually is more adequately described by a time-dependent rate coefficient, *k*(*t*), in such situations. To give an example, we compared recently intracellular trafficking of DHE and BChol in HeLa and BHK cells with normal and elevated fat content [26]. We observed that BChol is preferentially targeted to lipid droplets (LD’s) on a time scale of a few minutes, while DHE became much more enriched in the ERC and only to a minor extent transported to LD’s in those cells. The affinity of BChol for LD’s is likely a consequence of the attached BODIPY group, since BODIPY-dyes are used as prominent LD markers [66]. The bleaching kinetics of BChol was analyzed on a pixel-by-pixel basis using PixBleach and could be best described by a stretched exponential (StrExp) function, especially in LD’s [26,36]. The StrExp function, also called Kohlrausch function is an empirical decay law with broad use in photophysics for characterization of complex relaxation processes [67,68]. It is characterized by a time-dependent rate coefficient, *k*(*t*) instead of a rate constant, which is a consequence of a stochastic distribution of intrinsic bleach rate constants given by a probability density function (PDF) with a memory kernel. The differential equation for decay processes with underlying distribution of rate constants can be generally written as [67], see Equation (44):

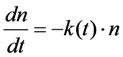
(44)
where *n* is the decaying species, for example number of fluorophores in a given volume with start amount *n*_0_. There exist many expressions for the rate coefficent, all leading to different decay laws (e.g., the Becquerel functions, also called compressed hyperbola or the Mittag-Leffler function) [69]. In case of the StrExp function, one derives the expression for the normalized decay law (*i.e.*, normalized to initial amount, *n*_0_, as Equation (45):

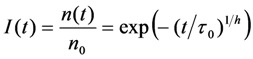
(45)


The formula for the rate coefficient becomes Equation (46):

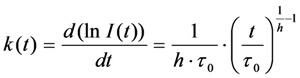
(46)


**Figure 7:**
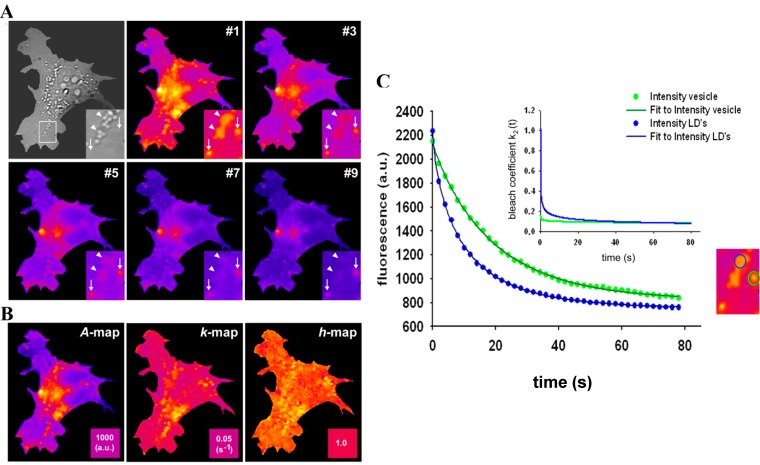
Stretched exponential photobleaching of BODIPY-cholesterol in lipid droplets. HeLa cells were labelled with BChol/MCD and imaged on a wide field microscope as described in Materials and Methods. Bleach stacks were fitted on a pixel-by-pixel basis to a StrExp function implemented in PixBleach. (**A**) most left panel shows bright field (Bf) image of cells with zoomed box indicating LDs (arrowheads) and position of endocytic vesicles, which do not give contrast in the Bf image (arrows). Selected frames of the bleach stack shown in the next left and following frames of panel (**A**) reveal preferred photobleaching of BChol in LD’s compared to the rest of the cells (see especially the inset, pointing to LD’s with arrowheads and slower bleaching vesicles with arrows); (**B**) shows from left to right the amplitude maps (‘A-map’), the map of fitted bleach rate constants, *k*_b_, (‘k-map’), and a map of the stretching parameters (‘h-map’); (**C**) fit of Equation (48) to a selected LD (blue symbols, data; blue line, fit) and vesicle (green symbols, data; green line, fit); the region selected is highlighted in the corresponding colour in the zoomed inset, which is the same, as in panel A. Inset of (**C**) shows time evolution of the bleach rate coefficient, as defined in Equation (47) using the fitting parameters.

The StrExp function is a generalization of the exponential function due to the stretching parameter *h*. For *h* > 1 the decay is faster than exponential for *t* < *τ* and slower afterwards. If the stretching parameter *h* = 1, one obtains the simple mono-exponential function. When fitting the StrExp function to bleach stacks of BChol labeled cells acquired on a wide field fluorescence microscope, we found that BChol bleaches significantly faster in LD’s than in other cellular areas (Figure 7 and [26]).

In addition, we observed stretched decays with *h* ~ 1.4–1.5 in LD’s, while in the rest of the cells, the photobleaching was only slightly stretched compared to a mono-exponential decay (*h* ~1.05–1.15) (see [26] and Figure 7B). This was not a consequence of a different fluorescence lifetime of BChol in LD’s. The fluorescence lifetime of BChol was bi-exponential with a major fraction contributing by ~80% and a mean fluorescence lifetime of *τ_f _*= 5.5 ns, well in accordance with model membrane studies [26,41]. The triplet yield of BODIPY fluorophores, as in BChol, has been shown to be very low [70]. A physical justification for using the StrExp function and thereby for a time-dependent rate coefficient could be the dependence of the bleaching process on a bleaching agent, for example the concentration of molecular oxygen, O_2_, or of generated singlet oxygen, ^1^*O*_2_, within the LDs [26,71]. Both, molecular oxygen and singlet oxygen catalyse photobleaching processes in tissues and in single cells, for example of GFP [72,73]. Also, oxygen is known to be highly soluble in very hydrophobic environments, like LD’s [74,75]. Another possible scenario for consumption of a bleaching agent could be the presence of a natural photosensitizer in the LDs. For example, triacylglycerol bearing polyunsaturated fatty acids or some vitamins could generate singlet oxygen after absorption of UV- and blue light [76]. This, in turn, could enhance photobleaching of droplet-localized sterols. To include this effect in our bleaching model, we can replace the intrinsic bleach rate constant by a rate coefficient according to Equation (47):

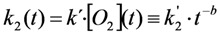
(47)


Thus, the rate coefficient replaces a bi-molecular rate process being dependent on a second species, which we assumed to be molecular oxygen. Alternatively, the rate coefficient could depend on some natural photosensitizer, as outlined above. No matter, what the second species actually is, it must be consumed during the reaction, such that the rate coefficient slows down in course of the bleaching process. The rate coefficient is defined as product of a new intrinsic bleach rate constant, 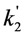, and a power law of time, in which the parameter *b* plays the same role as the stretching parameter, *h*, in the classical StrExp function (see Equations (45) and (46), above). We want to stress the point that time-dependent rate coefficients occur naturally in diffusion-limited bimolecular reactions, for example in fast fluorescence quenching [77]. The classical modelling approach for such processes is the Smoluchowski equation, in which the fluorescence decay becomes the sum of an exponential and a StrExp function, in which the stretching parameter is *b* = 0.5 [78]. Thus, we can further justify our approach by stating that diffusion of oxygen into the LD’s limits the overall bleaching rate.

Assuming that the REA is still valid, we can use Equation (47) in Equation (13) and find upon integration for the time evolution of *S*_1_(t), see Equation (48):

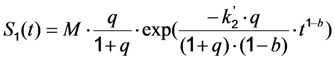
(48)


This function recovers for *b* = 0 Equation (14), and it can also well describe the measured photobleaching kinetics of BChol in various cellular areas (Figure 7C). We find a higher intrinsic bleaching rate constant of BChol in LD’s 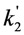 = 0.2742 s^−1^ and a much more stretched photobleaching decay (*b* = 0.2868 than in endocytic vesicles (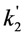 = 0.1145 s^−1^ and *b* = 0.0725; see legend to Figure 7 for other parameters). The bleaching rate coefficient, *k*_2_(t) slows down over time for both measured areas, but much more pronounced for LD’s. An interpretation could be that LD’s contain large amounts of (singlet) oxygen causing initially fast photo-oxidation of BChol, while over time oxygen gets depleted in LD’s. Assuming that replenishment from other cellular areas is slow compared to the bleach rate, oxygen availability would become rate limiting and thereby slow the photobleaching especially in LD’s. When incubating BHK cells overnight with DHE in complex with albumin, we found targeting of that sterol to LD’s as well, probably as a consequence of DHE esterification. Transport of DHE to LD’s after long time paralleled by its esterification has been found also in other cell types [79]. Interestingly, photobleaching kinetics of DHE were faster in LD’s than in other regions of BHK cells, and followed only in LD’s a stretched exponential decay (not shown). This further supports that LD’s provide a very special environment for photodestruction processes of hydrophobic fluorescent probes.

### 2.8. TiEm of the Non-Exponential Photobleaching Model

It remains to be determined, what the TiEm means in case of non-exponential photobleaching. We observed for many data sets that the goodness of fit, as judged by the χ^2^-value, is slightly better for a pixel-wise fit of the StrExp function compared to a fit with the mono-exponential model, while the respective time constant maps are comparable (not shown). These results suggest that the TiEm calculated as sum of pixel values from the reconstructed bleach stack of the StrExp fitting function can also correct for background artifacts and heterogeneous illumination across the field of view. To support this notion, we calculated the time integral of the acquired fluorescence for the model given in Equation (48). Since this is rather involved, we used Mathematica 7.0 (Wolfram Research Inc., Champaign, IL, USA) for that purpose, giving for 0 < *b* < 1, see Equation (49):

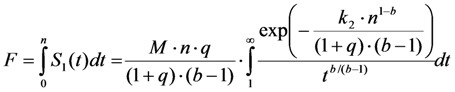
(49)


The integral term is called the exponential integral function. It can be expanded in a series to obtain approximate analytical expressions, or it can be analyzed numerically. Instead, we let *n* → ∞ and calculate directly the TiEm as Equation (50):

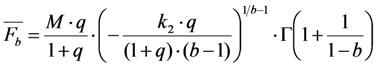
(50)
where 
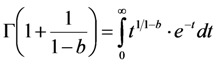
 is the Euler gamma function. 

**Figure 8:**
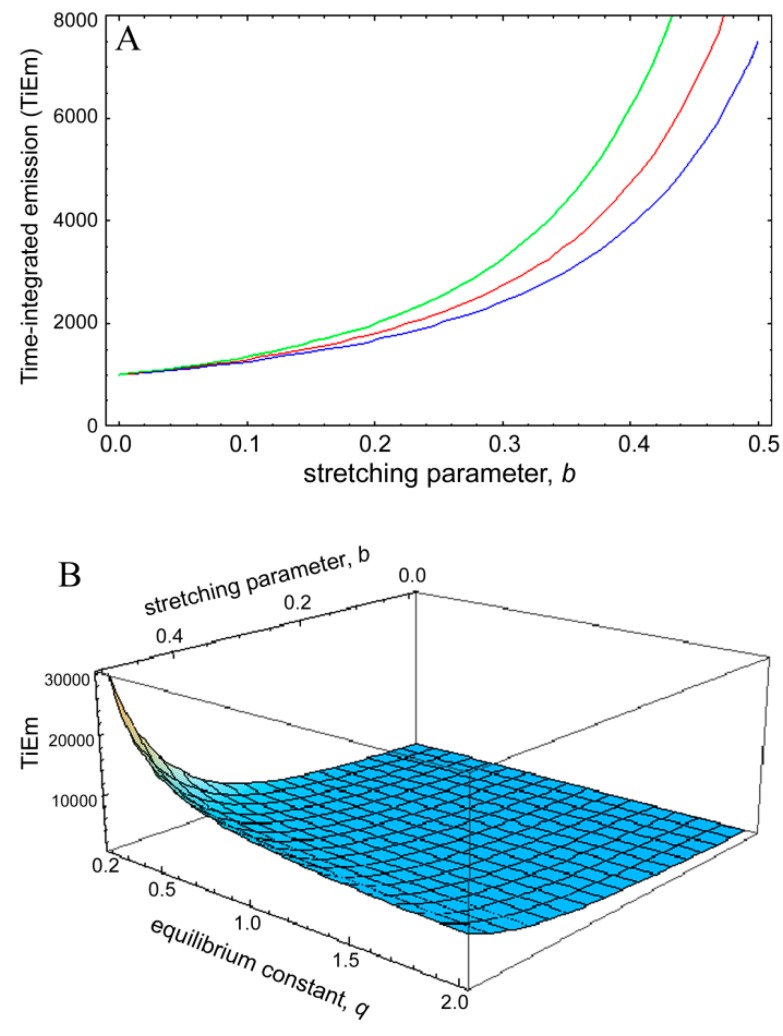
Simulation of time-integrated emission of bleaching kinetics with rate coefficients. The time integrated signal was calculated for the model in Equation (48) according to Equation (50) and plotted in Mathematica as a function of the stretching parameter, *b*, in this model (**A**). The bleach rate constant was *k*_2_ = 0.1 s^−1^, and *M* = 100 molecules corresponding to a TiEm from the singlet state (Equation (16)) of 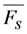 = 1,000. This equals the vsalue of 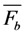 = 1,000 for *b* = 0. The TiEm for the model, 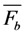, is plotted for three values of the equilibrium constant of the photocycle, *q* = 2 (blue curve), *q* = 1 (red curve) and *q* = 0.5 photons/molecule (green curve), respectively; (**B**) 3D plot of the same model with identical parameters but as function of both, *b* and *q*. See text for further explanations.

Clearly, for complex photobleaching kinetics with time-dependent rate coefficient the TiEm becomes dependent on the equilibrium constant of the photocycle, *q*. Thus, in this case, illumination intensity and fluorescence lifetime will affect the collectable signal of the bleaching fluorophores (*i.e.*, their total photon count number). To assess this quantitatively, the TiEm, for the bleaching model with rate coefficient (Equation (48)), as given in Equation (50), 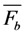, was plotted as function of the stretching parameter, *b*, for *M* = 100, *k*_2_ = 0.1 s^−1^ and various *q*-values (Figure 8). The curves in Figure 6A start at 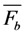 = 1,000 for *b* = 0 and thereby coincide with the TiEm of fluorescence with bleaching from the excited singlet state, 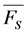, of Equation (16), above. For increasing values of *b*, we observe an increasing deviation of 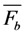 from 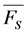 with (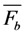
−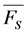) > 0, meaning that the TiEm is always larger in case of a rate coefficient than for constant intrinsic bleach rate constant (compare Equations (16) and (50)). Moreover, the difference is larger for small equilibrium constants between *S*_0_ and *S*_1_ than for large ones (e.g., compare *q* = 0.5 and *q* = 2.0; green and blue curve in Figure 8A or 3D plot in Figure 8B). 

Accordingly, for small deviations from a mono-exponential bleach process the pixel-wise fitting of a StrExp function to the fluorescence loss provides similar results as the mono-exponential fit. Also, even though the TiEm for the model with rate coefficient depends on illumination intensity and fluorescence lifetime of the probe via the parameter *q* (see Equation (50)), this dependence is low for small stretching parameters, *b*. The larger the *q*-values, implying high illumination intensities, the lower is the difference between 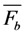 and 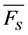 for a given value of the stretching parameter, *b*. Thus, in case of strong illumination, which will saturate the fluorophore, larger deviation of the bleaching kinetics from a mono-exponential decay can be tolerated to keep a meaningful interpretation of the TiEm (see Figure 8A,B). Together, bleaching models with underlying distributions of rate constants might be a suitable alternative to mono-and multi-exponential decay models. In some cases, a mechanistic underpinning for time-dependent rate coefficients is possible, while in other cases they purely serve the purpose of improving the fitting performance. For small deviations from the classical mono-exponential decay (*i.e.*, for narrow distribution of rate constants), the TiEm given by such models can be interpreted as shown for the mono-exponential decay model developed in this study. 

## 3. Experimental Section 

### 3.1. Reagents and Cell Labelling

Fetal calf serum (FCS) and DMEM were from GIBCO BRL (Life Technologies, Paisley, Scotland, UK). All other chemicals were from Sigma Chemical (St. Louis, MO, USA). C6-NBD-SM was purchased from Invitrogen/Molecular Probes Inc. (Life Technologies Europe BV, Naerum, Denmark). Buffer medium contained 150 mM NaCl, 5 mM KCl, 1 mM CaCl_2_, 1 mM MgCl_2_, 5 mM glucose and 20 mM HEPES (pH 7.4) as described [8]. A stock solution of DHE was made in ethanol and stored under nitrogen at −80 °C. For staining cells with sterol probes, a DHE or BChol labeling solution containing a complex of methyl-β-cyclodextrin with either DHE (DHE/MCD) or BChol (BChol/MCD) was generated as described [26]. Human skin fibroblasts (from Coriell Institute, Camden, NJ, USA) and HeLa cells were grown at 37 °C in an atmosphere of 5% CO_2_ until 90% confluence in complete DMEM culture medium supplemented with 1% glutamine, 1% penicillin and 10% FCS. The cells were placed on microscopy dishes and allowed to settle for another 48 h in the same growth medium. Fibroblast cells were labelled with DHE/MCD for 3 min, washed with buffer medium and chased for 1 h at 37 °C before imaging. HeLa cells were labelled with C6-NBD-SM after complexing this probe to defatted bovine serum albumin (BSA) as described previously for similar lipid probes [22]. Cells were pulse-labeled for 2 min at 37 °C, washed, chased in buffer medium for 30 min and imaged as described below. Alternatively, cells were labelled with BChol/MCD and chased as described for C6-NBD-SM.

### 3.2. Fluorescence Microscopy, Image Analysis and Simulation

Wide field epifluorescence microscopy was carried out on a Leica DMIRBE microscope with a 63 × 1.4 NA oil immersion objective (Leica Microsystems A/S Ballerup, Denmark) with a Lambda SC smartshutter (Sutter Instrument, Novato, CA, USA) as illumination control. Images were acquired either with an Orca 2 CCD camera (Hamamatsu Photonics, Hamamatsu, Japan) or with an Andor Ixon^EM^ blue EMCCD camera operated at −75 °C and driven by the Solis software supplied with the camera. DHE was imaged in the UV using a specially designed filter cube obtained from Chroma Technology Corp. (Bellows Falls, VT, USA) with 335-nm (20-nm bandpass) excitation filter, 365-nm dichromatic mirror and 405-nm (40-nm bandpass) emission filter. C6-NBD-SM and BChol were imaged using a standard fluorescein filter set [470-nm, (20-nm bandpass) excitation filter, 510-nm longpass dichromatic filter and 537-nm (23-nm bandpass) emission filter]. The illumination intensity was measured using a handheld power meter Orion/TH from Ophir Photonics (Har Hotzvim, Jerusalem, Israel) after calibration for UV light at a distance of 9 mm from the objective. For bleach stacks, images were acquired sequentially with acquisition time of 20 to 100 ms until probe fluorescence was faded away completely. Image stacks were analysed in ImageJ [37] using an upgraded version of the plugin PixBleach [80]. This upgrade allows the user to choose between varying ways of calculating the total intensity of a given bleach stack on a pixel-by-pixel basis (see Results section). Images with spatially varying intensity decay based on a given model were simulated using self-programmed Macros to ImageJ, as recently described [36,54]. 

## 4. Conclusions 

We present a detailed analysis of photobleaching processes of intracellular fluorophores in one-photon excitation microscopy. We discuss and model several photobleaching mechanisms and derive expressions for the bleaching kinetics and the TiEm. We show that the TiEm is in many experimentally relevant cases independent of illumination intensity and fluoresecence lifetime. Accordingly, the TiEm removes spatial variations in probe emission due to fluorescence saturation or local collisional fluorophore quenching in living cells. We provide several ways of calculating the TiEm including a new software solution as plugin to the popular ImageJ program. Determining the TiEm in this way from bleach stacks of labelled cells allows for correcting for illumination shading without need for fluorescent test layers, as done in earlier work [81,82]. We demonstrate that the TiEm also removes cellular autofluorescence, as long as the latter bleaches orders of magnitude slower than the probe fluorescence. Finally, we discuss photobleaching with time-dependent rate coefficients resembling a probability distribution of rate constants. This complements earlier studies by Koppel *et al.* using cumulant analysis of intracellular photobleaching [83]. We provide a concrete example of a stretched exponential decay of fluorescence of cholesterol analogs in LD’s and discuss the interpretation of the TiEm in this situation. We believe that the practical implementations of our analysis, especially for correcting of shading and autofluorescence will be of value for many applications. 

However, we also want to stress that our analysis is limited in two ways; first, it is restricted to two dimensions and does therefore not apply to photobleaching as it takes place in three-dimensional confocal microscopy [84]. Second, our model does not cover more complicated photobleaching processes. Examples for the latter are bimolecular photoreactions in the triplet state (either with oxygen or with semi-reduced or -oxidized radical forms of the dye molecule) [61], collisional triplet-triplet energy transfer between suitable dye molecules (e.g., as seen with porphyrins) [73], and excited state dimer formation, as known for pyrene [85,86]. It must also be emphasized that we model the effect of bimolecular bleaching processes through time-dependent rate coefficients (see Section 2.7 and Section 2.8, above), but do not consider explicitly the generation of singlet oxygen or free radicals, as for example known for porphyrins [73], for photoactivated GFP [72], and for intrinsically fluorescent sterols [87]. Neither is bleaching from the triplet or other higher excited states as consequence of absorbing a second photon (two-step photolysis) included in our analysis [48,88]. This phenomenon causes a quadratic increase of the empirical bleach rate constant, while our model always assumes saturation of the measured bleach rate constant for increasing excitation intensity. Two-step photolysis has been observed for various rhodamine and coumarine derivatives with high laser excitation beyond 10^4^ W/cm^2^, as used for single-molecule spectroscopy [48,59,88,89]. It is relevant in confocal imaging and fluorescence correlation spectroscopy (FCS), where the laser is focused to a diffraction-limited spot giving very high irradiance within the focal volume [59,90]. The exact intensity threshold above which 2-step photolysis takes place is a dye-specific property, and might be also relevant for membrane-bound triplet probes, like anthracene [91]. In widefield-illumination, the photon flux during irradiation is much lower, since the excitation light is spread over the whole field of view [32,92]. Both, widefield and confocal imaging can be optimized to achieve a linear relationship between irradiance of excitation and extent of photobleaching [92]. Given suitable lasers as excitation source, however, the irradiance in widefield-illumination can be tuned up, such that a quadratic dependence of the measured photobleaching rate constant on excitation intensity is found. For example, evidence for 2-step photolysis was obtained above 20 mW illumination power focused on a 100 × 100 μm area for rhodamine-dyes embedded into polyvenyl alcohol (PVA) [89]. We have measured the excitation power for our wide field setup being equipped with a 100 W mercury arc lamp (see 3. Experimental Section, above for details). From that, we calculated excitation intensities of 0.256 mW/cm^2^ and 0.585 mW/cm^2^ for excitation wavelengths of 335-nm (20-nm bandpass) and 470-nm (20-nm bandpass), respectively, which are focussed by the objective to an area of approx. 100 × 100 μm in the focal plane. Thus, irradiation intensities used here for demonstrating the practical implementation of our analysis are well below those known to cause 2-step photolysis [48,88]. Reversible photobleaching, as known for rhodamine, fluorescein and carbocyanine fluorophores [93] as well as for color variants of GFP [94], is another phenomenon not included in our model. It is observed, though, at excitation intensities well above those used in our study but can, for example, compromise fluorescence recovery after photobleaching (FRAP) experiments intended for measuring intracellular protein dynamics [94,95,96]. Finally, we did not take reverse intersystem crossing leading eventually to delayed fluorescence into account, nor did we include photoselection in our analysis [97,98,99]. It will be an interesting future project to include such effects as well as more complicated photobleaching processes into our model. 

## Supplementary Materials

Supplementary materials can be accessed at: http://www.mdpi.com/1420-3049/19/8/11096/s1.

## Supplementary Files

Supplementary File 1
