# Supplementary material for: Photobleaching Kinetics and Time-Integrated Emission of Fluorescent Probes in Cellular Membranes
Source: Molecules. 2014 Jul 29;19(8):11096–130. doi: 10.3390/molecules190811096 (PMC6271172; doi:10.3390/molecules190811096)

Supporting Information

The complete kinetic model shown in Equation (19) was solved numerically using a Runge-Kutta scheme implemented in Mathematica 7.0 (Wolfram research Inc., Champaign, IL, USA) using the same parameters as shown in Figure 4 and the accompanying text but varying intersystem crossing rate constants (from $k_3 = 6.6 \times 10^2\ s^{-1}$ to $k_3 = 6.6\ s^{-1}$) and decay rate constant from the triplet state (from $k_5 = 50\ s^{-1}$ to $k_5 = 0.5\ s^{-1}$), thereby keeping the triplet yield unchanged. We found increasing deviation of the analytical model (black lines being almost constant around $S_1 \sim 8$) from the complete numerical simulation of Equations (28)–(30) despite constant triplet yields of $q_T = 660/50 = 13.2$, cyan line; $q_T = 66/5 = 13.2$, pink line and $q_T = 6.6/0.5 = 13.2$, blue line). Thus, using the REA fails to describe the initial bleaching kinetics, if the bleach rate constant from the triplet state is only about 500 fold slower than the kinetics of transition to and from the triplet state.

Figure S1. The extended photobleaching model for slow intersystem crossing.

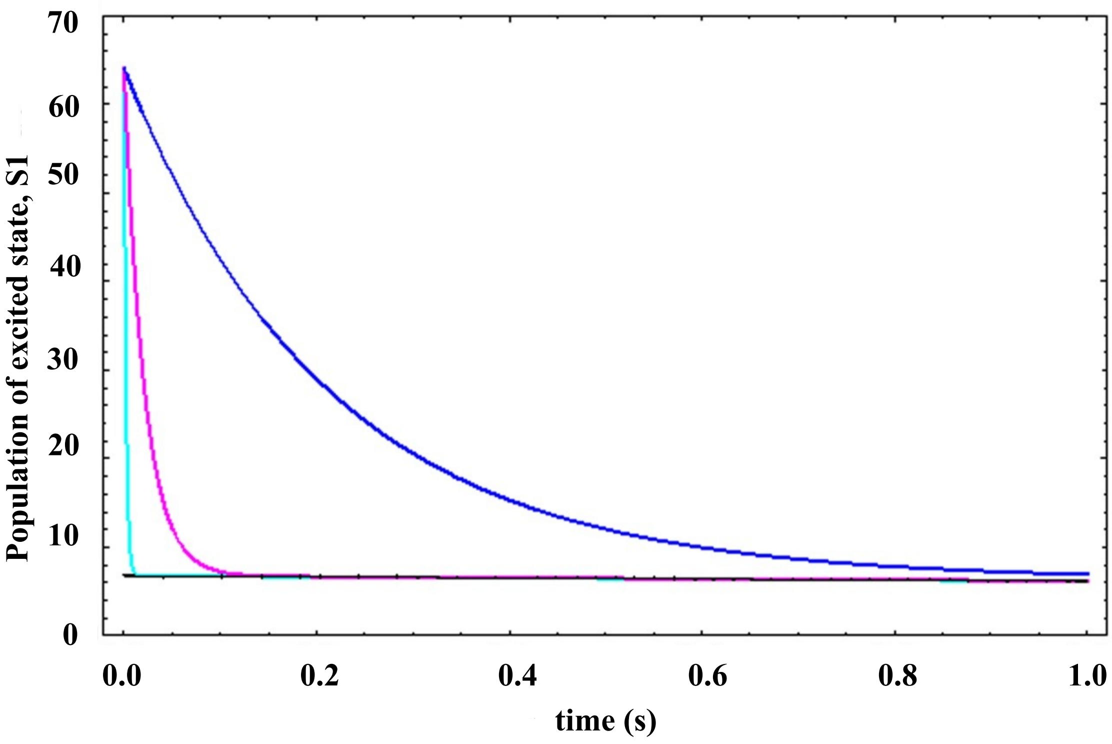

Supplement: Supplementary File 1 [file molecules-19-11096-s001.pdf]
